# Compound Heterozygous ATM Variants Cause Adolescent‐Onset Cerebellar and Extrapyramidal Disease Without Telangiectasia in a Consanguineous Pakistani Family

**DOI:** 10.1155/genr/5644954

**Published:** 2026-06-25

**Authors:** Faiza Aslam, Weizhen Ji, Lauren Jeffries, Anant Wadhwa, Monica Konstantino, Muhammad Noman, Nigel S. Bamford, Emily Mis, Saquib A. Lakhani, Sadaf Naz

**Affiliations:** ^1^ School of Biological Sciences, University of the Punjab, Lahore, Pakistan, pu.edu.pk; ^2^ Pediatric Genomic Discovery Program, Department of Pediatrics, Yale University School of Medicine, New Haven, Connecticut, USA, yale.edu; ^3^ Department of Pediatrics, Cedars Sinai Guerin Children’s, Los Angeles, California, USA; ^4^ Department of Neurology, Yale University School of Medicine, New Haven, Connecticut, USA, yale.edu; ^5^ Departments of Pediatrics and Molecular and Cellular Physiology, Yale University School of Medicine, New Haven, Connecticut, USA, yale.edu; ^6^ Department of Neurology, University of Washington, Seattle, Washington, USA, washington.edu

**Keywords:** ataxia–telangiectasia, ATM, consanguinity, Pakistan, reverse phenotyping

## Abstract

Ataxia–telangiectasia (A–T) is a heterogeneous genetic disorder with a recessive mode of inheritance resulting from biallelic variants in the A–T mutated gene (ATM). Besides ataxia, the disorder involves compromised immunity and an increased risk of malignancies. We recruited a consanguineous Pakistani family with multiple individuals having adolescent‐onset ataxia. Phenotyping and clinical testing were completed for the patients. DNA samples from multiple individuals were used for bidirectional exome sequencing at 100X coverage, and data were aligned to the hg19 genome assembly. Sanger sequencing was completed to confirm the segregation of the variants. Multiple sequence alignments of orthologous proteins from diverse species were performed using ClustalO to check the amino acid conservation. Patients in the family manifested gait and limb ataxia, postural instability, and dystonia. A known heterozygous pathogenic nonsense ATM variant, c.2413C > T, p.(Arg805Ter), in trans with a new unreported missense variant, c.8708C > T, p.(Pro2903Leu), was identified, which segregated with the disease. The missense variant affected an amino acid, which was conserved in evolution. Telangiectasia of the eyes and skin was absent in the affected individuals, which led to the initial misdiagnosis of the disease as cerebellar ataxia. There were no reports of malignancies in the family, and affected individuals were alive in their third and fourth decades of life. Thus, molecular analyses resulted in the reclassification of the disease as A–T, an example of reverse phenotyping. The study expands the phenotypic heterogeneity of A–T and extends the allelic spectrum of ATM variants.

## 1. Introduction

*Ataxia–telangiectasia mutated* (ATM) gene located on chromosome 11q22‐23 contains 63 exons [1] and encodes a serine/threonine kinase, a member of phosphoinositide 3‐kinase‐related kinases. ATM phosphorylates several proteins, which are involved in maintaining cellular homeostasis. It also plays vital roles in the DNA damage response, cell cycle checkpoints, and apoptosis [2]. Biallelic variants of ATM are associated with A–T (OMIM#208900), a rare autosomal recessive disorder. These pathogenic variants lead to a complete or partial loss of protein function. A–T is a multisystem disorder and is notable for causing neurologic impairment, including ataxia, oculomotor apraxia, chorea, peripheral neuropathy, difficulty with speech and swallowing, as well as symptoms and signs of extrapyramidal disease [3–5]. Telangiectasias, which are small, dilated blood vessels, are classically found in the skin and conjunctivae. Moreover, impaired ATM protein function causes chromosomal instability that, along with defective DNA damage repair, leads to a high risk of malignancies, which are primarily leukemias and lymphomas [6]. Studies have shown that ATM has a role in the redox reactions of cells, and a deficiency of the protein is associated with oxidative stress due to the accumulation of reactive oxygen species [7].

Gene variants leading to a complete lack of ATM kinase activity result in classic A–T with severe, early‐onset disease around 2 years of age, and most patients die around 30 years of age due to malignancies and sinopulmonary infections. However, mutations which leave some residual kinase activity result in variant A–T with a later onset of disease and a milder phenotype. With a slower progression of disease, individuals with variant A–T live into the fourth decade and beyond [8].

The prevalence of A–T is 1 in 40,000 to 1 in 100,000 live births [5]. Diagnosis for A–T relies on clinical correlation by expert clinicians, and it may help in the disease management as the individuals can be treated with antibiotics or immunoglobulin replacement therapy for the respiratory infections [9, 10]. Patients also should avoid unnecessary radiation exposure due to their cancer predisposition, and the type of variant also indicates course of treatment if cancer develops. ATM variant identification also helps with carrier counseling and prenatal screening [11]. Apart from the patients, carriers of pathogenic or likely pathogenic heterozygous ATM variants also have a significantly increased risk of developing cancers [12].

Populations with higher consanguineous marriages have a greater likelihood of developing recessively inherited genetic disorders, due to increased homozygosity [13]. Studies in these populations can lead to genetic discoveries and may also help in identifying genotype–phenotype correlations [14, 15]. In this study, we utilized whole exome sequencing (WES) to evaluate a consanguineous Pakistani family having multiple individuals affected with progressive ataxia. We describe the finding of a previously unreported ATM variant in trans with a known pathogenic variant as a cause of adolescent‐onset variant A–T, with neither dilated blood vessels nor telangiectasias in the eyes and conjunctiva.

## 2. Materials and Methods

### 2.1. Enrollment of Family and Clinical Characterization

This study was approved by the Institutional Review Boards of the School of Biological Sciences, University of the Punjab (IRB#00005281, FWA 00010252), and Yale University, School of Medicine (HIC#1411014977). The proband was identified from the Punjab province of Pakistan, followed by obtaining the family and clinical history in 2018. Both parents, four patients, and two unaffected siblings provided written informed consent to participate in the study. Blood samples were collected from all participants. The patients were videotaped according to a standard videotaping protocol [16]. An assessment of the patient’s neurological condition was determined post hoc by neurologists in the United States. The videos were assessed in a blinded manner by Drs Anant Wadhwa and Nigel S. Bamford. The degree of ataxia was rated using a modified Scale for the Assessment and Rating of Ataxia (SARA) [17]. For the SARA ratings, the patients’ videos were examined for the assessment of gait, sitting, speech disturbance, and the nose–finger test, and a weighted kappa was calculated for inter‐rater agreement. The video recordings did not allow for objective assessment of stance, finger chase, and fast alternating hand movements.

### 2.2. Molecular Analyses

DNA was extracted from whole blood by standard methods [18]. WES was performed using IDT xGen exome capture followed by Illumina HiSeq 4000 sequencing at Yale YCGA under a research protocol in 2020. Paired‐end sequence reads were converted to FASTQ format and were aligned to the reference human genome (hg19). The variants were called using GATK and annotated using AnnoVar [19]. Variants were further selected on the basis of frequencies in global populations, family inheritance patterns, and their pathogenicity predictions by multiple software tools. Meta‐predictors such as Combined Annotation‐Dependent Depletion (CADD) or rare exome variant ensemble learner (REVEL) were also used, while possible effects on splice sites were assessed using SpliceAI, AbSplice, NNPredict, and ASSP. Frequencies of all shortlisted variants were also checked in the exome data (100X coverage with at least 20 mean on‐target depth) of ethnically matched 300 controls from the Punjab province. Copy number variant (CNV) analysis was performed with our institutional custom pipeline using the normalized exome depth of coverage from the same batches of submitted samples. DNA samples of all the remaining family members were analyzed for the identified variants by Sanger sequencing. Laboratory tests of serum alpha fetoproteins (AFPs), carcinoembryonic antigen (CEA), and immunoglobulins A and E were performed for the proband after molecular analyses of affected individuals. The patients declined imaging studies. Conservation matrix for the partial orthologous proteins was determined with the analysis of Jensen–Shannon divergence (JSD) using ConsCapra07 (v1.1.0) (https://bio.tools/conscapra07). Evolutionary conservation of the identified variant was also observed with GeneBe (https://genebe.net/). All data analyses were done with descriptive statistics, and power calculations are not relevant to this study.

## 3. Results

### 3.1. Clinical Synopsis

A consanguineous family from the Punjab province of Pakistan with multiple affected individuals displaying clinical symptoms of ataxia and postural instability was identified and recruited for the study. The proband (V:2; Figure 1A) was a 47‐year‐old female who developed ataxia and postural instability at 15 years of age, with symptoms progressively worsening over time. She reported occasional difficulty in breathing characterized by subjective shortness of breath, though she did not have a history of frequent respiratory tract infections. She also did not report any skin abnormalities. She was a well‐kept, thin‐appearing female of approximately normal stature. Her feet were inverted at rest, and she demonstrated high arches and hammer toes. There were no dysmorphic features or visible telangiectasia on the skin or conjunctiva. Neurological examination was notable for severe axial and appendicular ataxia, generalized dystonia and extrapyramidal signs, including generalized dystonia, striatal toes, and impassive face. She conversed and answered questions appropriately. Her speech was fluent. Her eyes appeared well‐aligned. She demonstrated a near‐persistent, impassive facial expression, with frequent procerus activation, but would smile at times. Cervical dystonia was evident with a clear, fixed leftward tilt of the neck. Spooning was noted with hands outstretched. Dystonic flexion was present at multiple joints of the fingers and toes. Dysmetria and ataxia were evident in both arms on the finger‐to‐nose test. She demonstrated marked titubation, truncal swaying, and an ataxic gait, necessitating holding on to adjacent objects for stability when walking. Titubation of the head and torso persisted in the sitting position. She had at least antigravity muscle strength in the lower extremities and was able to squat well without assistance. Striatal toes with spontaneous hallux extension were observed during ambulation. Tendon reflexes could not be formally assessed due to the video examination.

**FIGURE 1:**
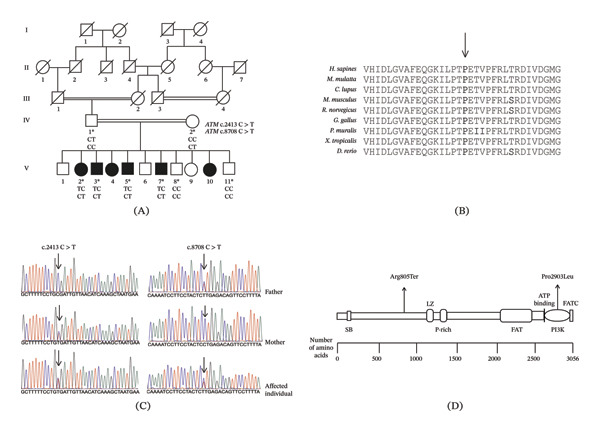
Patients’ information, amino acid conservation, electropherograms, and diagrammatic representation of the protein. (A) Pedigree of the recruited family RDFA14. Asterisks indicate the individuals who participated in the study. Genotypes of the participants are mentioned with their respective symbols (wild‐type allele CC and heterozygous allele CT) in the pedigree. DNA samples of individuals IV:1, IV:2, V:2, and V:7 were used for whole exome sequencing. Patients did not provide consent to perform the MRI analysis. (B) Conservation of p.Pro2903 residue is depicted among most diverse orthologues. This residue is conserved in all lineages of vertebrates. (C) Partial electropherograms of father, mother, and a patient are shown from the relevant *ATM* exons. Complementary DNA sequences are provided. Arrows indicate the bases substituted which led to the nonsense and missense *ATM* variants. The father is heterozygous for the c.8708C > T variant, while the mother harbored the c.2413C > T *ATM* variant in the heterozygous form. Affected individuals are compound heterozygous for both variants. (D) Diagrammatic representation of the ATM protein. N‐terminus has a substrate binding (SB) site, a leucine zipper (LZ), and a proline‐rich domain, while the C‐terminus contains the FAT (FRAP–ATM–TRRAP) and PI 3‐kinase‐like domain and FATC (FAT–C‐terminal domain). Region of the ATM protein affected by both *ATM* variants identified in the RDFA14 family is indicated with arrows.

Multiple other individuals in the proband’s family also developed similar symptoms between 12 and 16 years of age, consisting of progressively worsening ataxia, postural instability, lack of coordination, oculomotor apraxia, and similar extrapyramidal signs and symptoms (Table 1). Following the assessment of SARA, gait, sitting, speech, and nose–finger tests were rated to be significantly affected. The calculations of weighted kappa showed that there was substantial agreement between the two raters (Supporting Tables 1 and 2). Inter‐rater reliability was high, with an intraclass coefficient (ICC) of 0.75.

**TABLE 1:** Clinical findings with both common and atypical manifestations of ataxia telangiectasia.

Patient ID and clinical features	Proband (individual V:2)	Affected sibling (individual V:3)	Affected sibling (individual V:5)	Affected sibling (individual V:7)
Age in 2024 (years)	51	48	44	41
Sex	Female	Male	Male	Male
Worsening gait and limb ataxia	++++	++++	+++	+++
^∗^Telangiectasia of eyes and skin	Absent	Absent	Absent	Absent
Extrapyramidal symptoms	Focal dystonia, striatal toe, impassive facial expression	Focal dystonia, impassive facial expression	Focal dystonia	Focal dystonia
Respiratory infections	Mild infections	No	No	No
^∗^Immunological problems	No	No	No	No
^∗^Malignancies	No	No	No	No
^∗^Infertility	No	No	No	No

*Note:* ++++ = severe phenotype, +++ = milder phenotype.

^∗^These conditions are frequently observed in patients with *ATM*‐related ataxia.

None of the affected individuals had dermal or conjunctival telangiectasias. All family members, with ages ranging from 37 to 47 years (in 2021), also denied a history of respiratory infections or cancer. No one reported breathing difficulties except for the proband. The serum levels of immunoglobulins A and E tested for the proband were in the normal ranges.

### 3.2. Molecular Analyses Reveal Two Likely Pathogenic Variants in ATM

DNA was obtained from the proband, both her biological parents, three affected siblings, as well as two unaffected siblings (Figure 1A). Two affected and three unaffected siblings did not consent to provide samples. WES was performed for the proband (V:2), her affected male sibling (V:7), and their parents (IV:1 and IV:2) with a mean coverage ranging from 85X to 145X. Both patients had two rare compound heterozygous variants in *ATM* (NM_000051.3): maternally inherited c.2413C > T, p.(Arg805Ter), a known pathogenic variant (ClinVarVCV000216021.8), and paternally inherited c.8708C > T, p.(Pro2903Leu), a novel likely pathogenic variant (ClinVar accession VCV001048073) (Table 2). The Pro2903 residue is highly conserved (Figure 1B), and the p.(Pro2903Leu) variant is absent from most public databases, including 1000 genomes, EPS6500, and gnomADv2, but is present in gnomADv4 with an allele frequency of 0.000001239. Neither variant was found in the exome data of 300 control individuals (600 chromosomes). Sanger sequencing in the additional available family members confirmed segregation with the disease phenotype (Figure 1C). Despite known consanguinity, no shared rare (defined as present in fewer than 1% of individuals in any subpopulation) likely deleterious homozygous variants were identified in the data of the patients.

**TABLE 2:** Identified disease‐causing gene variants in the patients of family RDFA14.

Gene	Genomic location	HGVS c.DNA	HGVS protein	Zygosity	Parent of origin	CADD	ACMG	First report
ATM	Chr11: 108129749 (GRCh37)	NM_000051 c.2413C > T (VCV000216021.8)	p.(Arg805Ter)	Heterozygous	Mother	35	P	PMID: 12552559
ATM	Chr11: 108224529 (GRCh37)	NM_000051 c.8708C > T (VCV001048073)	p.(Pro2903Leu)	Heterozygous	Father	32	LP	This study

*Note:* ACMG = American College of Medical Genetics and Genomics, CADD = Combined Annotation‐Dependent Depletion score (those above 20 have a high chance of being damaging), P = pathogenic, PMID = PubMed identifier.

Abbreviations: HGVS, Human Genome Variation Society; LP, likely pathogenic.

The frequency of the variant p.(Arg805Ter) is 0.00003981 (gnomADv2) or 0.000008056 (gnomADv4), observed in both South and East Asian, as well as European populations. This nonsense variant was classified as “pathogenic” (PVS1, PM2, PP1, PP5) according to the American College of Medical Genetics and Genomics (ACMG) clinical variant classification guidelines [20]. Similarly, the c.8708C > T, p.(Pro2903Leu) variant, was designated as “likely pathogenic” (PM2, PM3, PP1, PP3) according to the ACMG guidelines. Additionally, multiple *in silico* analyses (e.g., scale‐invariant feature transform (SIFT), polymorphism phenotyping (PolyPhen), functional analysis through hidden Markov models (FATHMM), and meta‐analytic support vector machine (MetaSVM) predicted that the missense change p.Pro2903Leu has a deleterious effect with CADD score = 34 and REVEL pathogenicity score = 0.887. The c.8708C > T, p.(Pro2903Leu) variant was not predicted by multiple software to disrupt the splicing of the transcript. The variant c.8708C > T, p.(Pro2903Leu), was very well conserved with a JSD score of 0.842 and PhyloP100 of 7.9. No significant findings were obtained on performing CNV analysis, and the ATM coding region also displayed a normal copy number.

Following an identification of the biallelic ATM variants, the proband underwent lab tests screening for potential malignancies, including a serum AFP level of 30 μg/L (normal range 0–10 μg/L) and a serum CEA level of 1.01 ng/mL (normal range 0–2.5 ng/mL).

## 4. Discussion

We report results from a successful WES analysis for a consanguineous family from Pakistan. Affected individuals in this family had a history of adolescent‐onset ataxia as the primary clinical feature, consistent with a diagnosis of variant A–T, including postural instability, a perception of muscle weakness, and difficulty with coordination that reportedly worsened with increasing age. Despite the consanguinity, we identified compound heterozygous ATM variants: a previously unreported variant, c.8708C > T, p.(Pro2903Leu), classified as likely pathogenic, in *trans* with a known pathogenic variant, c.2413C > T, p.(Arg805Ter).

The adolescent‐onset, milder symptomatology of variant A–T in the current family suggests the possibility for residual activity of the ATM mutant protein, though it remains to be determined experimentally. The truncating variant located in Exon 16 encoding the N‐terminal domain (Figure 1D) is predicted to cause non–sense‐mediated decay of mRNA, or loss of function of ATM due to the production of a nonfunctional truncated protein. It has previously been categorized pathogenic both as a homozygous variant and as a heterozygous variant, mostly in trans with other truncating *ATM* variants in A–T patients with the classic form of ataxia, and presumably results in the absence of functional protein [21, 22]. The p.(Pro2903Leu) missense variant, located in the protein kinase domain (Figure 1D), results in the substitution of a polar uncharged amino acid by a nonpolar aliphatic amino acid. Additionally, the native proline at 2903 is a highly conserved amino acid (Figure 1B). Taken together with the findings of extreme rarity of this variant in normal population databases, as well as a high CADD score, the predicted deleterious nature according to multiple *in silico* predictors and perfect segregation with the phenotype in the family, further strengthen the suggestion that this variant is pathogenic. We hypothesize that the p.Pro2903Leu ATM protein variant likely leaves some residual kinase activity, given the milder symptoms and late onset seen in the patients. Functional studies in the future could establish this experimentally. Previous studies demonstrate that ATM kinase activity was preserved in milder A–T due to compound heterozygous missense *ATM* variants (p.Leu2541Arg) and (p.Leu1046Phe) detected in three patients of a family [23].

While patients with A–T classically develop telangiectasias and early‐onset ataxia, members of this family presented with a unique constellation of adolescent‐onset cerebellar and extrapyramidal disease with no evidence of telangiectasias. The absence of telangiectasias is rare, but has been noted previously in A–T individuals with milder disease due to missense variants [4, 23–25]. Previous studies have also reported that frameshift and nonsense variants, leading to null alleles, cause more severe disease phenotypes than missense and splice site variants. The latter may be hypomorphic alleles with some residual kinase activity [10]. Varied disease courses, such as we report here, may result in misdiagnosis or delayed diagnosis of A–T [26]. A–T misdiagnosis has been reported in various studies due to a delay in the onset of symptoms, and a recent study demonstrates that the cardinal features of the disease were absent in a patient having atypical A–T, due to the presence of a null allele and a missense allele. The patient participating in that study presented mild symptoms of infant‐onset generalized chorea and skin abnormalities, including café au lait spots, axial freckling, and hypopigmentary lesions across the trunk and extremities. However, telangiectasia was absent [27].

The level of AFP observed for the proband in this study was highly elevated (30 μg/L) as per the normative values used by the local diagnostic laboratory. Previous studies have shown that the levels of AFP are generally elevated in most individuals presenting A–T, but few individuals with a milder form of A–T exhibit levels of AFP within normal range [3, 9, 28]. However, a recent retrospective cohort study analyzing the medical records of individuals with A–T found elevated levels of serum AFP for all patients exhibiting either classic A–T or variant A–T [29]. There was no history of malignancies or sinopulmonary infections in this family despite multiple affected individuals still living in their fourth decade of life or older. A previous study has shown that patients carrying missense variants of *ATM* with residual kinase activity had no history of recurrent infections, and the development of cancer was also delayed [23]. Immunodeficiency is also an important indicator of the A–T disease. However, it is reported in only 1/3 of the patients with *ATM* variants [30]. If present, immunogenic deficiencies lead to high incidences of respiratory defects and severe disease prognosis in A–T patients [31]. Only the proband in our family had breathing difficulties, which was not correlated to an immunogenic deficiency, since her immunoglobulin levels were within the acceptable ranges. Gonadal function appears to be intact within this family, as three of the affected individuals have children. Gonadal atrophy is a common feature of classic A–T, while individuals with milder forms of A–T are usually fertile [5].

Although the neurological symptoms and signs can vary widely among A–T patients, findings in siblings are often similar [4]. Primary dystonia due to variant A–T with homozygous variants of the *ATM* gene has been described, but such patients lack telangiectasias, ataxia, and cerebellar atrophy on imaging [32]. Dopa‐responsive cervical dystonia with *ATM* variants has also been described [33]. Though these patients may develop telangiectasias, ataxia is not found. Two siblings of Indian descent were reported to have intellectual disability, conjunctival telangiectasias, ataxia, choreoathetosis, ocular movement abnormalities, axial myoclonus–dystonia, and peripheral neuropathy [34]. In another study involving a Pakistani consanguineous family, the youngest 4‐year‐old child had no symptoms of the disease, though he was homozygous for the same *ATM* nonsense variant that causes severe classic A–T in his older siblings [35].

Currently, there is no definitive treatment for A–T and patients are treated to improve the disease symptoms, including recurrent infections and cancers. A recent study found that patients carrying *ATM* variants with or without kinase activity respond differently to cancer treatment therapies. Patients carrying *ATM* variants with absent kinase activity have higher treatment‐related mortality and lesser event‐free survival as compared to individuals carrying *ATM* variants with residual ATM kinase activity. Hence, patients without ATM activity can receive less intensive therapy to reduce the fatal toxicity of cancer drugs, while individuals with residual ATM activity can receive near‐standard therapy for treatment of cancer. This study underscores the protective nature of even the residual activity of the ATM kinase against the toxicity of cancer drugs [36].

### 4.1. Strengths and Limitations of This Study

WES helped in the correct diagnosis of disease in this family as A–T that was initially misdiagnosed as cerebellar ataxia. The study broadened the genetic spectrum of *ATM* with the identification of a new missense disease‐causing variant. With the help of this diagnosis, disease counseling was provided to patients to refrain from direct prolonged sunlight exposure, and information regarding the future risk of developing malignancies was discussed. A limitation of our work was that we were not able to determine the residual kinase activity due to the missense ATM variant.

## 5. Conclusion

In conclusion, additional sequencing of South Asians and other underrepresented populations will increase our knowledge about variants, both common and rare, in different groups, which will assist in the identification of benign and pathogenic alleles. It is hypothesized that despite the predicted deleterious nature of the novel missense variant that we identified, the ATM protein is possibly left with some residual activity of its kinase function, which seems to have resulted in a milder form of A–T and a longer life span in the affected individuals. This study highlights the phenotypic spectrum of ATM gene and identifies another variant likely to result in the variant A–T phenotype.

**Nomenclature**
A–T: Ataxia–telangiectasia
WES: Whole exome sequencing
ATM: Ataxia–telangiectasia mutated gene
AFP: Alpha fetoprotein
CEA: Carcinoembryonic antigen
CADD: Combined Annotation‐Dependent Depletion
PolyPhen: Polymorphism phenotyping
SIFT: Scale‐invariant feature transform
REVEL: Rare exome variant ensemble learner
FATHMM: Functional analysis through hidden Markov models
MetaSVM: Meta‐analytic support vector machine
ACMG: The American College of Medical Genetics and Genomics
HGVS: Human Genome Variation Society
LP: likely pathogenic

## Author Contributions

Faiza Aslam performed experimental work and wrote the manuscript. Lauren Jeffries, Monica Konstantino, Weizhen Ji, and Emily Mis analyzed data, helped with clinical characterization, and critically reviewed the manuscript. Anant Wadhwa and Nigel S. Bamford reviewed the video recordings, clinically characterized the affected individuals, and contributed to writing the manuscript. Muhammad Noman identified the family and provided samples and clinical data. Sadaf Naz and Saquib A. Lakhani planned and supervised the work, analyzed data, and contributed to writing the manuscript.

## Funding

This work was funded by the Higher Education Commission of Pakistan and Yale New Haven Hospital.

## Ethics Statement

This study was approved by the Institutional Review Boards of the School of Biological Sciences, University of the Punjab (IRB#00005281), and the Yale University School of Medicine (HIC#1411014977).

## Consent

Written informed consents were obtained from all the participants. Consent for publication of any identifying information was obtained from the participants.

## Conflicts of Interest

Saquib A. Lakhani is a part‐owner of Victory Genomics, a startup company unrelated to this work. The other authors declare no conflicts of interest.

## Supporting Information

Additional supporting information can be found online in the Supporting Information section.

## Supporting information


**Supporting Information** Supporting data contain the assessment of the SARA scores by two raters for phenotypes of the patients (Supporting Table 1) and the analysis of the SARA data and calculation of the weighted kappa (*κ*) (Supporting Table 2).

## Data Availability

The *ATM* variants described in this family were submitted to ClinVar (https://www.ncbi.nlm.nih.gov/clinvar/) with the following accession numbers VCV001048073 and VCV000216021.8. Additional data may be obtained upon reasonable request by correspondence with the authors.
