# Supplementary material for: Compound Heterozygous ATM Variants Cause Adolescent‐Onset Cerebellar and Extrapyramidal Disease Without Telangiectasia in a Consanguineous Pakistani Family
Source: Genet Res (Camb). 2026 Jun 25;2026:5644954. doi: 10.1155/genr/5644954 (PMC13305135; doi:10.1155/genr/5644954)
Supplement: Supplementary file 1 — Supporting Information Supporting data contain the assessment of the SARA scores by two raters for phenotypes of the patients (Supporting Table 1) and the analysis of the SARA data and calculation of the weighted kappa (κ) (Supporting Table 2). [file GENR-2026-5644954-s001.docx]

**Supplementary Table 1: Assessments of SARA scores by two raters**

| **Item** | **Proband**  **(Individual V:2)** | | **Affected Sibling**  **(Individual V:3)** | | **Affected Sibling**  **(Individual V:5)** | | **Affected Sibling**  **(Individual V:7)** | | **SARA Scoring** |
| --- | --- | --- | --- | --- | --- | --- | --- | --- | --- |
|  | **Rater 1** | **Rater2** | **Rater1** | **Rater2** | **Rater 1** | **Rater 2** | **Rater 1** | **Rater 2** |  |
| Gait | 6 | 7 | 6 | 6 | 4 | 3 | 3 | 3 | **Range 0-8**  0: Normal  8: unable to walk with support |
| Sitting | 2 | 2 | 1 | 1 | 1 | 2 | 1 | 1 | **Range 0-4**  0: Normal  4: Unable to sit for 10s without continuous support |
| Speech | 2 | 2 | 2 | 2 | 2 | 2 | 2 | 2 | **Range 0-6**  0: Normal  6: Speech unintelligible |
| Nose-Finger | 3 | 3 | 3 | 3 | 3 | 3 | 3 | 3 | **Range 0-4**  0: Normal  4: Unable to perform 5-pointing movements |

**Supplementary Table 2: Analyses of the SARA scores and calculations of weighted kappa (κ)**

| **SARA Item** | **Neurologist 1 Mean ± SD** | **Neurologist 2 Mean ± SD** | **Weighted kappa score (κ)** |
| --- | --- | --- | --- |
| Gait | 4.75 ± 1.50 | 4.75 ± 2.06 | 0.79 |
| Sitting | 1.25 ± 0.50 | 1.50 ± 0.58 | 0.50 |
| Speech | 2.0 ± 0 | 2.0 ± 0 | 1.00 |
| Nose-Finger Test | 3.0 ± 0 | 3.0 ± 0 | 1.00 |
